# Implementing Cognitive Stimulation Therapy (CST) for Dementia in a Low-Resource Setting: A Case Study in Tanzania Exploring Barriers, Facilitators, and Recommendations for Practice

**DOI:** 10.1007/s43477-024-00142-6

**Published:** 2025-01-11

**Authors:** Emily Fisher, Sarah Mkenda, Jessica Walker, Ssenku Safic, Charlotte R. Stoner, Catherine Dotchin, Stella-Maria Paddick, Godrule Lyimo, Jane Rogathi, Maria Jelen, Matthew Breckons, Jane Fossey, Richard Walker, Aimee Spector

**Affiliations:** 1 Research Department of Clinical, Educational and Health Psychology, University College London, 1-19 Torrington Place, London, WC1E 7HB UK; 2 Sheffield Institute for Translational Neuroscience, University of Sheffield, Sheffield, UK; 3 Department of KCRI, Kilimanjaro Christian Medical College, Moshi, Tanzania; 4 Population Health Sciences Institute, Newcastle University, Newcastle upon Tyne, UK; 5 Mount Meru Regional Hospital, Arusha, Tanzania; 6 School of Human Sciences, University of Greenwich, London, UK; 7 Department of North Tyneside General Hospital, Northumbria Healthcare NHS Foundation Trust, Newcastle upon Tyne, UK; 8 AGE Research Group, Translational and Clinical Research Institute, Newcastle University, Newcastle upon Tyne, UK; 9 Faculty of Medical Sciences, Newcastle University, Newcastle upon Tyne, UK; 10 Faculty of Health and Life Sciences, University of Exeter, Exeter, UK

**Keywords:** Psychosocial, Intervention, Consolidated Framework for Implementation Research, CFIR, Low-and-middle Income Country

## Abstract

Cognitive Stimulation Therapy (CST) is a group psychosocial intervention for people with mild-to-moderate dementia. Despite evidence supporting its effectiveness, cost-effectiveness, and cultural adaptation internationally, CST has yet to be implemented in routine practice outside of the UK. This study consisted of multiple phases. In the first phase, we engaged stakeholders to explore the barriers and facilitators to CST implementation in Tanzania. In the second phase, we developed implementation strategies. In the third phase, we tested these strategies in a study of CST in a tertiary hospital in northern Tanzania. The Consolidated Framework for Implementation Research (CFIR) guided the study. We conducted stakeholder engagement with decision-makers, healthcare professionals, people with dementia and their family caregivers (*n* = 49) to identify barriers and facilitators to implementation. We developed an implementation plan that included 20 implementation strategies related to 12 CFIR constructs. Subsequently, we tested these strategies by running eight CST groups with 49 participants. In follow-up interviews with people with dementia, carers and healthcare professionals, we identified 18 key CFIR constructs as barriers or facilitators to successfully implementing CST. CST was compatible with the standards of care in a tertiary referral hospital in northern Tanzania. To implement CST in low-resource settings, we recommend running dementia awareness initiatives, screening for dementia in outpatients and community settings, developing a train-the-trainer model, employing a task-shifting approach, and creating a dementia-friendly space for dementia services. Our findings can inform future efforts to implement CST and other psychosocial interventions for dementia in low-resource settings.

**Supplementary Information:**

The online version contains supplementary material available at 10.1007/s43477-024-00142-6.

## Implementing Cognitive Stimulation Therapy for Dementia in a Low-Resource Setting: A Case Study in Tanzania

Dementia presents a significant global health challenge, affecting over 55 million people worldwide. This figure is projected to nearly double every 20 years, with most of the increase in prevalence in low-and middle-income countries (World Health Organization, 2021). Dementia has an estimated annual global cost of US $1313.4 billion (Wimo et al., 2023). Despite the majority of people with dementia worldwide living in low-and-middle-income countries, nearly three-quarters of the global spending occurs in high-income countries (Wimo et al., 2023). The highest increases in cases are expected in sub-Saharan Africa, North Africa and the Middle East, mainly due to population ageing and population growth (Nichols et al., 2022). Studies in rural Tanzania estimated an age-adjusted prevalence of dementia of 6.4% in people aged 70 and above in 2009-10, which increased to 8.9% in 2018-19 (Longdon et al., 2013; Yoseph et al., 2021).

This increase in prevalence presents critical challenges in countries across sub-Saharan Africa, which face a high disease burden driven by (1) communicable diseases, (2) injury and trauma, and (3) a rise in non-communicable conditions (Guerchet et al., 2017). These countries’ health and social care systems are fragile and under-resourced, and they struggle to deal with this triple burden (Guerchet et al., 2017). In particular, there is a large treatment gap for mental, neurological and substance misuse disorders in low-income countries (Chibanda et al., 2020). There is a shortage of specialist healthcare staff, such as psychiatrists, psychologists and occupational therapists, neurologists and geriatricians, to diagnose and support the increasing number of people with dementia across all low-income countries (Guerchet et al., 2017). A 2015 survey by The World Health Organization (WHO) estimated that in Tanzania, there were 0.056 psychiatrists and 0.009 psychologists per 100,000 people working in the mental health sector (WHO, 2019)

Lack of healthcare support, combined with limited dementia awareness and high levels of stigma, culminate in a significant burden of caregiving, which falls upon family members (Mwendwa et al., 2022; Walker et al., 2023). Many people turn to traditional healers, faith healers or alternative medicine for treatment (Hindley et al., 2017; Mushi et al., 2014). Additionally, many individuals do not recognise dementia as a medical condition; instead, they may perceive it as a normal part of ageing or a condition caused by witchcraft or ‘God’s punishment’ (Brooke & Ojo, 2020; Hindley et al., 2017).

Access to pharmacological treatment for dementia across sub-Saharan Africa remains a significant challenge (Akinyemi et al., 2022). Commonly prescribed anti-dementia drugs such as cholinesterase inhibitors are not on the WHO Essential Medicines List (WHO, 2023). Although being on this list does not guarantee protection from supply shortages, which are frequent in Tanzania (Wales et al., 2014), their inclusion would be an important step towards improving access to these drugs (Tang et al., 2023). When cholinesterase inhibitors are unavailable, people with dementia may be prescribed antipsychotics, which are associated with severe side effects (Tang et al., 2023).

Non-pharmacological or psychosocial approaches offer an opportunity for the management of dementia in Tanzania. Unlike medications, these approaches would not suffer from supply shortages, but they do need personnel. However, many psychosocial interventions do not require specialist equipment or knowledge, lending themselves to a ‘task-shifting’ approach (Shahmalak et al., 2019; Spedding et al., 2014). With this approach, the healthcare specialists typically deliver is transferred to non-specialist teams who receive training and supervision from specialist teams (Joshi et al., 2014; Prince et al., 2016).

### Cognitive Stimulation Therapy for Dementia

Cognitive Stimulation Therapy (CST) is a group psychosocial intervention for people with mild-to-moderate dementia, delivered twice weekly over seven weeks. It comprises themed activities, which engage participants in a social group environment through tasks such as physical activity, word association and discussions (Spector et al., 2003). CST was developed in the UK and has been adapted and evaluated globally. A systematic review of 36 randomised controlled trials (Woods et al., 2023) reported moderate-quality evidence for a small short-term benefit to cognition (standardised mean difference [SMD] 0.40, 95% confidence interval [CI] 0.25–0.55). Secondary outcomes in a smaller number of studies showed moderate-quality evidence for a slight increase in self-reported quality of life (SMD 0.23, 95% CI 0.07–0.42) and high-quality evidence for a benefit to communication and social interaction (SMD 0.53, 95% CI 0.36–0.7). It has been deemed cost-effective in England (Knapp et al., 2022).

CST has previously been translated and culturally adapted for use in Tanzania and Nigeria (Mkenda et al., 2018). Key adaptations included modifying cultural references and colloquialisms, adapting activities to accommodate low literacy levels and uncorrected sensory impairment, and changing the recommended materials and equipment to those readily available in low-resource settings (Mkenda et al., 2018). The adaptation followed the Formative Method for Adapting Psychotherapy (Hwang, 2009), which ensured that the core components of CST remained consistent.

A stepped-wedge trial of the adapted CST programme conducted in rural Tanzania (*n* = 34) showed that there were improvements in cognition in the immediate start group compared to the delayed start group (F = 7.08, *p* = 0.01) (Paddick et al., 2017a, b). This was a small-scale feasibility study, so the results are exploratory. However, a review of culturally adapted interventions for dementia suggested that interventions that are effective in initial randomised controlled trials remain effective in different settings as long as the intervention is feasible, acceptable, and the core components are retained (James et al., 2021).

CST is proven effective and cost-effective and has been adapted internationally to various cultures. Despite this evidence, CST is yet to be implemented widely in routine practice outside the UK. This reflects the implementation gap between research and practice, which has been well-defined in dementia care (Lourida et al., 2017; Vernooij-Dassen & Moniz-Cook, 2014). The 2011 World Alzheimer’s Report advocated using CST as a low-cost and effective intervention in low- and middle-income countries and recommended its routine adoption (Prince et al., 2011). A key recommendation from The Africa Dementia Consortium is translating research evidence into practice and policy (Akinyemi et al., 2022).

### Implementation Science Framework

We used the Consolidated Framework for Implementation Research (CFIR) to explore the implementation of CST. This enables comparisons of barriers and facilitators in different contexts and for other interventions (Damschroder et al., 2022). The CFIR is a widely-used determinant framework that includes factors known or hypothesised to impact implementation outcomes. The framework consists of 48 constructs and 19 sub-constructs across five domains: (1) Innovation characteristics; (2) Outer setting; (3) Inner setting; (4) Characteristics of individuals involved in implementation, categorised into their need for the intervention, their capability, availability and motivation (based on the COM-B model [Michie et al., 2011]); and (5) Implementation processes. The CFIR was initially developed in 2009 with 39 constructs across five domains and has been used and evaluated in low-and-middle-income country settings (Damschroder et al., 2009; Means et al., 2020). Recommendations were made for refinement for use in low-and-middle-income country settings, which were implemented in an updated CFIR in 2022 (Damschroder et al., 2022; Means et al., 2020).

This study took place within the CST-International research programme (Spector et al., 2019). The specific objectives for Tanzania were: (1) to explore barriers and facilitators to implementing CST in Tanzania; (2) to develop and appraise strategies for implementing CST at a hospital in northern Tanzania; and (3) to make recommendations for ongoing and sustainable CST provision that can be applied more broadly in low-resource settings.

The study was approved by the Kilimanjaro Christian Research Ethics and Review Committee (certificate number 2245) and the Tanzanian National Institute for Medical Research (NIMR) Ethics Committee (reference NIMR/HQ/R.8a/Vol.IX/ 2714).

## Methods

**Fig. 1:**
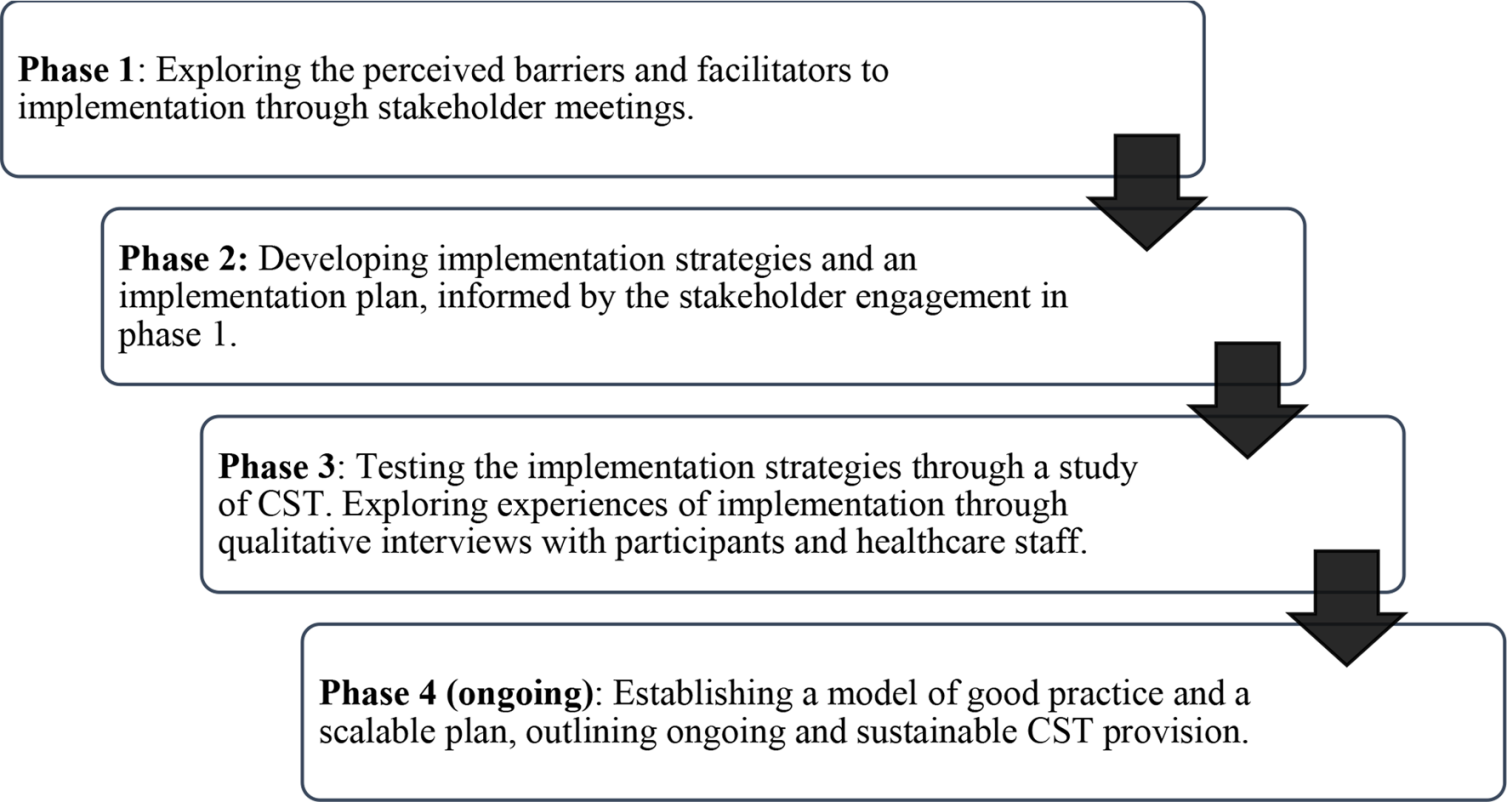
Four Study Phases

This was a mixed-methodology study with four overlapping phases, as outlined in Fig. 1. The Tanzania programme team consisted of clinicians, nurses, occupational therapists, psychologists, and researchers from Tanzanian government healthcare facilities, UK higher education institutions, and healthcare trusts.

Phase 1 and 2 methodology for the three countries involved in the CST-International project has been outlined in more detail in a previous publication (Stoner et al., 2020). Here, we present a brief overview of Phases 1 and 2 in Tanzania.

### Phase 1. Stakeholder Meetings

We held stakeholder meetings to explore perceived barriers and facilitators to CST implementation with three distinct stakeholder groups: (1) decision makers such as service managers and heads of department; (2) healthcare professionals who would deliver CST in their role; and (3) people with dementia and their family caregivers.

#### Recruitment and Initial Meetings

We recruited decision-makers and healthcare professionals from governmental healthcare institutions in Moshi and Arusha in northern Tanzania. We recruited people with dementia and carers who had previously been involved in a pilot study of CST from both the outpatient caseload and the community. The international research team developed questions iteratively based on CFIR constructs (Damschroder et al., 2009), and we tailored interview guides for different stakeholder groups.

Stakeholders discussed perceived barriers and facilitators to CST implementation. Meetings with decision makers and healthcare professionals were conducted in English and Kiswahili, and the meeting with people with dementia and carers took place in Kiswahili.

#### Compilation of Barriers, Facilitators and Implementation Strategies

Following initial meetings, the research team compiled the identified barriers and facilitators and proposed implementation strategies to overcome the obstacles and support facilitators. These were agreed upon through discussion.

#### Ranking of Implementation Strategies

The international team and stakeholders independently rated the proposed strategies according to perceived importance (essential, desirable, advisory) and ease of execution (easy, intermediate, difficult). Ratings were quantified and the mode was calculated to prioritise the implementation strategy.

### Phase 2. Development of Implementation Plan

The international team developed an implementation plan summarising the barriers, facilitators and implementation strategies identified in Phase 1. These strategies were organised by CFIR construct. We prioritised the strategies with the highest modes and reached a consensus on implementation strategies through discussion within the project team. The plan includes strategies that were considered essential and those for further consideration, depending on the availability of sufficient resources.

### Phase 3. Testing Implementation Strategies with a Study of CST

The implementation strategies were tested through a pre-post study of CST in a hospital outpatient department in Moshi, Northern Tanzania. The study aimed to recruit between six and eight CST groups, totalling approximately 50 people with dementia.

#### Inclusion Criteria and Recruitment

Inclusion criteria for CST groups were informed by previous studies of CST (Paddick et al., 2017b; Spector et al., 2003): (1) meeting the ICD-11 criteria for dementia (WHO, 2019), evaluated by a trained clinician; (2) having mild to moderate dementia, rated on the Clinical Dementia Rating Scale (Morris, 1997); (3) having adequate hearing and vision to follow conversations and comment on visual material; (4) able to participate in a group for one hour; (5) willing and able to complete measures of cognition and quality of life; and (6) willing and able to travel to attend CST sessions.

We used two recruitment strategies. Firstly, patients with a confirmed dementia diagnosis from the hospital psychiatry department caseload who met inclusion criteria were invited to attend CST groups. Occupational therapists, nurses and research assistants were also trained to screen outpatients aged 60 and over at diabetes and hypertension clinics using the IDEA 6-item cognitive screen (Gray et al., 2014). This measure has a scoring range of 0–15, with lower scores indicating greater cognitive impairment. Patients who scored ten or below were invited to assessment by a psychiatrist, and those who received a dementia diagnosis were invited to CST groups. Researchers received written informed consent from people with dementia and their carers. Those unable to read and write indicated consent through a thumbprint.

#### CST Intervention

All eligible individuals were invited to participate in 14 CST sessions over seven weeks. Groups comprised 5–8 people with dementia and two group leaders. Group leaders were occupational therapists trained by a ‘master trainer’ who had received training from the International CST Centre at University College London. Additionally, a nurse was present to assist with patient care, and a cleaner helped with room preparation. The group leaders delivered sessions according to the Tanzanian CST manual (Mkenda et al., 2018).

#### Implementation Outcomes

Implementation outcomes, adapted from Proctor et al., 2011, included acceptability and feasibility, which were explored through stakeholder interviews (see *Qualitative interviews).* We further explored acceptability by evaluating the attrition rate, average number of sessions attended, and rate of outcome measurement completion. Penetration was assessed according to recruitment of the target sample of 50 participants, the number of groups run, the number of staff trained to deliver CST and the number of staff trained as CST trainers. We calculated the target sample of 50 pragmatically based on available time and resources to run enough CST groups to explore feasibility, acceptability and implementation issues.

#### Cost of CST

We collected the costs of running CST training and CST sessions. This included staff time and associated costs, equipment and refreshments, and participant transport reimbursement.

#### Outcome Measures

Participants with dementia completed three standardised measures before and after the intervention: (1) The Alzheimer’s Disease Assessment Scale–Cognitive Subscale (ADAS-Cog): a 21-item measure of cognitive function (Rosen et al., 1984), adapted for use in Tanzania (Paddick et al., 2017a, b); (2) The World Health Organization Quality of Life - Brief Version (WHOQOL-BREF): a 26-item measure of the quality of life developed for use in low-and-middle-income countries, addresses quality of physical health, psychological health, social relationships and environment (The WHOQOL Group, 1998); and (3) IDEA study Instrumental Activities of Daily Living (IDEA-IADL): an 11-item questionnaire developed for use in Tanzania (Collingwood et al., 2014).

Family caregivers completed four measures: (1) The Zarit Burden Interview (ZBI), a widely used 22-item self-report measure of strain and stress (Zarit et al., 1980); (2) Dementia Caregiver Experience Scale (DemCarES): a 17-item measure to assess stress and strain developed for use in low-resource settings (personal communication by Vaitheswaran, 2023); (3) Client Service Receipt Inventory (CSRI): used to collect information on participants’ economic circumstances, and pre-and post-intervention healthcare use and expenditure (Beecham & Knapp, 2001); and (4) The Resource Utilisation in Dementia-Lite Version (RUD-Lite): collects data on formal and informal care resource use (Beecham & Knapp, 2001).

The ZBI is one of the best available measures of caregiver burden, though there are concerns about its cross-cultural validity and that it underestimates burden in low-resource settings (Brinda et al., 2014). To overcome this, the DemCarES measure was added to the battery of outcome measures.

These outcome measures are consistent with those used in previous studies of CST (Woods et al., 2023). The main objective was to assess the feasibility and acceptability of collecting these data for CST implementation. Measures were either already available in Kiswahili or were translated and back-translated by bilingual team members. Assessments were carried out by trained nurses, occupational therapists and research assistants who had not been involved in the participants’ CST groups. Data were at first collected using paper forms. After the first two CST groups, the data collection process was changed to using Open Data Kit (ODK)^®^ software to limit missing data. This open-source mobile data collection platform can be used offline on tablets, and data collectors were provided training.

#### Semi-Structured Interviews

After the CST groups were completed, semi-structured interviews took place with individual participants from two distinct stakeholder groups: (1) people with dementia and their family members, and (2) healthcare professionals (including CST group leaders, psychiatrists and nurses involved in screening and implementation support). The interviews aimed to explore factors related to the acceptability of the intervention, the feasibility of implementing the intervention, and perceived barriers and facilitators to implementation.

The international research team developed interview guides according to the CFIR constructs (Damschroder et al., 2009) and tailored them to each stakeholder group. The topic guides covered experiences and perceptions of the CST programme and implementation strategies, key barriers and facilitators to effective implementation, and recommendations for future practice leaders (see supplementary material).

Two researchers (one from the UK team and one from the Tanzanian team) who had not been involved in delivering the CST programme led interviews with people with dementia. This was done to minimise response bias. Interviews with people with dementia and their carers were conducted in Kiswahili, and interpretation was provided for the UK researcher. Interviews with healthcare professionals were conducted in English and led by a UK researcher.

#### Qualitative Data Analysis

Interviews were recorded with consent and transcribed. Before analysis, two bilingual researchers with experience in qualitative research translated transcripts in Kiswahili into English. A member of the research team (JW) checked the translations and made clarifications with the translator. Analysis was carried out by three researchers: a health services researcher, a medical student and an undergraduate psychology student (EF, JW and MJ). All three researchers had received training in qualitative methods and supervision from experienced qualitative researchers (MB and JF).

First, the three researchers read the transcripts to familiarise themselves with the data. We used a combined inductive and deductive approach using NVivo^®^. We coded the data using inductive thematic analysis (Braun & Clarke, 2019). Then, we mapped the themes onto the updated CFIR as a deductive framework (Damschroder et al., 2022). This approach allowed us to capture themes not covered by the CFIR. Coders met regularly to ensure they approached the data in a similar way. Any discrepancies were resolved through discussion.

## Results

### Phase 1. Stakeholder Meetings

Stakeholder meetings took place in 2018 in Arusha and Moshi. A total of 49 stakeholders attended, including Group 1: decision-makers including directors of services and heads of departments at local hospitals, and regional mental health coordinators (*n* = 5); Group 2: healthcare professionals including nurses, doctors, counsellors, psychologists and physiotherapists (*n* = 33); and Group 3: people with dementia and carers (*n* = 11).

Through discussion, stakeholders created an initial list of barriers and facilitators. The international project team then organised these into a table. The team suggested ways to overcome the obstacles and support the facilitators. A subset of the stakeholders (*n* = 12, including one decision maker, eight healthcare professionals, and three people with dementia and carers) and four project team members ranked these proposed strategies.

### Phase 2. Implementation Plan

Table 1 presents the highest-ranked implementation strategies from the action plan alongside their corresponding CFIR constructs. To support implementation, the international team developed a checklist of barriers and facilitators and a guidance document for newly trained CST group leaders (see supplementary material).

**Table 1:** Actions from implementation plan for CST Delivery in Moshi, Tanzania

CFIR construct and domain	Barrier/ facilitator identified	Proposed actions to support implementation	Rating
Outer setting			
Local attitudes	Barrier: Dementia stigma and misconceptions.	Develop a Dementia Awareness Course for carers.	Essential
	Barrier: Perception of dementia as a natural part of ageing and not seen as a medical problem.	Include information on the underlying pathology of neurodegenerative diseases in the Dementia Awareness Course.	Further consider
Critical incidents	Barrier: Rainy season will make some roads unpassable.	Consider the rainy season’s impact on groups and delaying groups if appropriate.	Essential
Policies and laws	Facilitator: CST should be included in treatment guidelines.	Explore opportunities to develop national guidelines on dementia treatment.	Further consider
Inner setting			
Structural characteristics; Work infrastructure	Barrier: Lack of cascading training model, with only one available trainer.	‘Master trainer’ course for CST group leaders to be developed to increase the pool of trainers. Tanzania-specific adaptions made to the ‘master training course’.	Essential Essential
	Barrier: Staff are often very busy and may not have time to run CST groups.	Organise and invite newly qualified professionals to regular CST training. Inform hospital staff that group leaders need protected time and ensure permission is granted.	Essential Essential
Available resources; Funding/Materials and equipment	Barrier: Funds may not be available for purchasing CST equipment/ refreshments.	Hospital management contacted to discuss replacing CST equipment if damaged/lost after the study finishes.	Essential
Access to knowledge and information	Barrier: Minimal teaching on dementia in medical schools.	Contact higher education institutes to discuss including CST in taught programmes.	Essential
Individuals			
High-level leaders Opportunity / Motivation	Facilitator: Need for authorisation at organisational level.	Invite hospital management/ policymakers to take the Dementia Awareness Course.	Further consider
Innovation recipients; Need	Barrier: Visual impairment of people with dementia may impact engagement.	If visual impairment is detected during CST screening, refer the individual to an ophthalmologist.	Further consider
Innovation recipients; Opportunity / Capability	Barrier/facilitator: Some older people receive free healthcare, but this is very variable.	Prominent insurance companies should be contacted to discuss whether CST could be covered.	Further consider
Process			
Assessing needs; Innovation deliverers	Facilitator: Training and support for group leaders.	All CST group leaders should be given a local checklist and asked to complete this checklist before running their first CST group.	Essential
Assessing needs; Innovation recipients	Barrier: Long travel time for a 45-minute session.	Outcomes collected during the feasibility assessment include a question on how the attendees travelled to the group.	Further consider
Planning	Barrier: Dementia stigma and misconceptions.	Organise Dementia Awareness Courses for family carers and deliver at regular intervals.	Essential
Tailoring strategies	Barrier: People may live far from health facilities. Public transport is unreliable. Some roads are untarmacked. Travel time for a 45-minute session is long.	Agree on whether local or hospital settings are required for each group and agree on travel arrangements.	Essential
		Group leaders to agree with carers how often the group should run (once or twice weekly).	Further consider
Engaging; Innovation recipients	Barrier: Lack of awareness of available services.	Engage with local media to advertise CST.	Essential
	Barrier: Village events/ market days may prohibit attendance.	Enumerators may be contacted to identify market days/ likely interruptions to CST groups	Further consider

Note. The research team developed the actions informed by stakeholder engagement activities. We have since reviewed the original action plan and adjusted the CFIR constructs based on the update by Damschroder et al., 2022, to ensure consistency throughout the reporting of the study phases. The actions have not changed, and the updated constructs can be linked to the original ones (Damschroder et al., 2009)

### Phase 3. Study of CST

The CST sessions scheduled to begin in Moshi in 2020 were disrupted by the COVID-19 pandemic, delays in obtaining ethical approval, and the rainy season. Screening for CST sessions started in November 2021, and CST groups took place in Moshi between March and September 2022. CST sessions were delivered in the hospital psychiatry department by occupational therapists trained as CST group leaders.

#### Screening and Recruitment

**Fig. 2:**
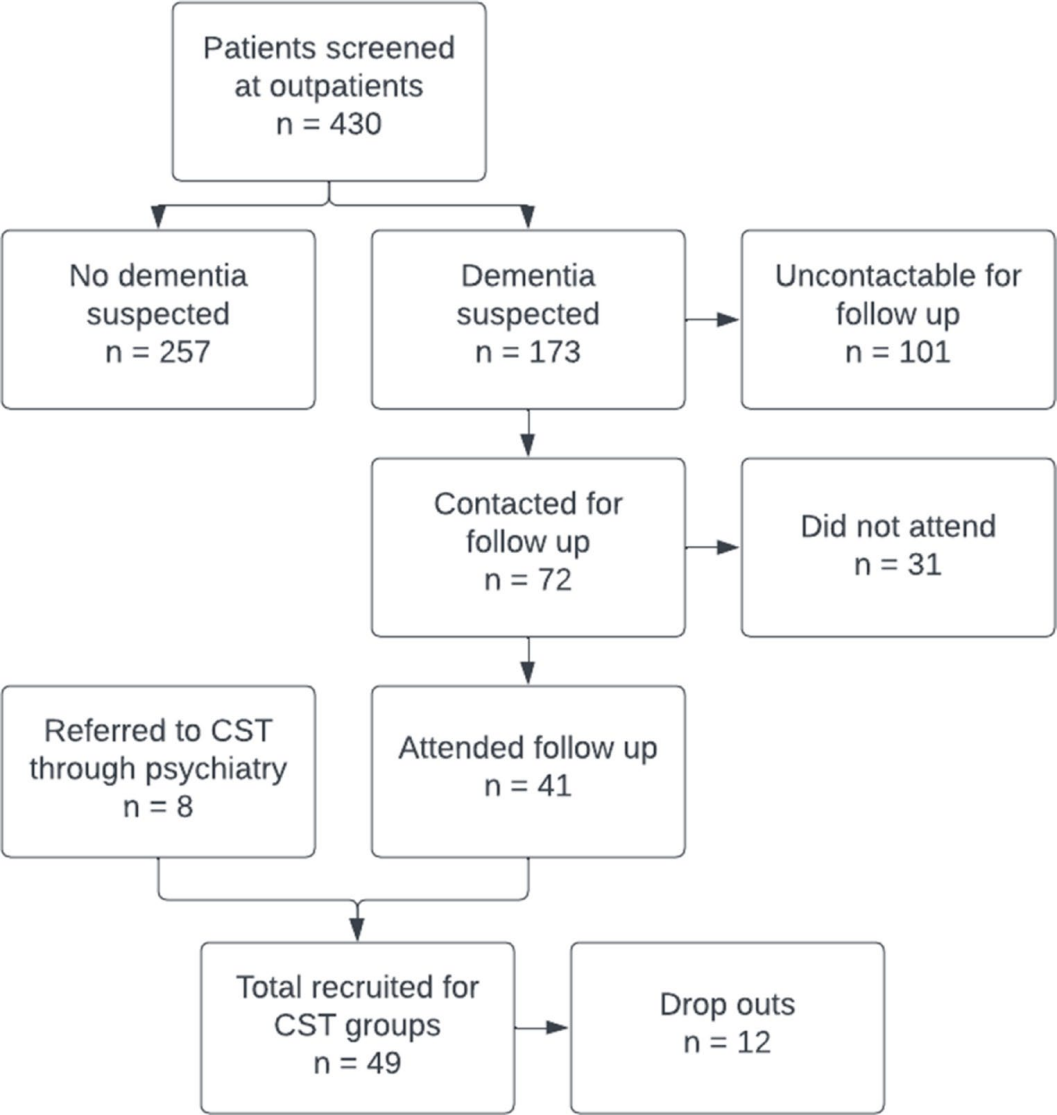
Screening, recruitment and attrition

A total of 430 patients were screened for dementia at hospital outpatient clinics. Dementia was suspected in 173 cases who scored below the cut-off on the IDEA cognitive screen. However, only 41.6% (*n* = 72) were contactable to organise a follow-up diagnostic appointment. There was a high rate of non-attendance for follow-up appointments (43.1%). All those who attended follow-up appointments accepted the offer of attending CST groups. Figure 2 outlines the screening and recruitment process.

#### Recruitment and Attendance Outcomes

A total of 49 participants were recruited across eight CST groups. Table 2 shows the sociodemographic details of the people with dementia and their carers. Baseline data were missing for 8 participants. There were 12 dropouts across the groups, and one group had to be disbanded as 3 out of 6 participants dropped out. The remaining three participants were invited back to attend another group. Overall attendance was 81%. Reasons for non-attendance included illness and carer commitments.

**Table 2:** Demographic information of CST participants and carers

Characteristic	*n*	%
*Person with dementia*		
Sex ^a^		
Female	15	36.6
Male	26	63.4
Tribe ^a^		
Chagga	36	87.8
Pare	4	9.8
Shambaa	1	2.4
Marital status ^a^		
Married	25	61
Divorced	3	7.3
Widowed	11	26.8
Single	1	2.4
Other	1	2.4
Primary language ^a^		
Kiswahili	32	78
Kichagga	8	19.5
Pare	1	2.4
Religion ^a^		
Christian	37	90.2
Muslim	4	9.8
Education level ^a^		
Completed Primary	16	39
Completed Secondary	8	19.5
Completed Tertiary	12	29.3
Minimal	3	7.3
None	2	4.9
*Carer*		
Sex ^a^		
Female	30	73.2
Male	11	26.8
Education ^a^		
Completed Primary	18	43.9
Completed Secondary	15	36.6
Completed Tertiary	8	19.5
Work status ^a^		
Homemaker (full-time)	6	14.6
Paid full-time work	10	24.4
Paid part-time work, not salaried	10	24.4
Retired	3	7.3
Student	2	4.9
Unemployed	10	24.4
Living with person with dementia ^a^		
Yes	27	65.9
No	14	34.1
Relationship to person with dementia ^a^		
Son/Daughter	20	48.8
Spouse	10	24.4
Sibling	4	9.8
Son-in-law/Daughter-in-law	3	7.3
Friend	2	4.9
Another relative	2	4.9

Note. *N* = 49. People with dementia were on average 72.4 years old (SD = 8.9 years) and carers were on average 50.0 years (SD = 15.7 years) [*n* = 39]

^a^ Data missing for *n* = 8

Seven staff members attended CST training, including four occupational therapists, two occupational therapy trainees and one nurse. Three occupational therapists were already trained CST group leaders, so this acted as a refresher course. They were subsequently trained as CST trainers in the ‘master trainer’ programme. Additionally, nine staff members received training in screening for dementia and delivering outcome measures.

A culturally tailored Dementia Awareness Course (DAC) was developed (Stoner et al., 2022), which was delivered to around 50 hospital staff. DAC sessions were also run in community settings for 25 carers and four people with dementia for carers and people with dementia who had attended CST sessions.

#### Cost of CST and Staffing

The cost of CST sessions per group (assuming 14 sessions with seven participants) was 1,884,857 Tanzanian Shillings (TZS) (710 US Dollars), of which 48.3% were staff costs, and 33.4% were patient transport reimbursement costs. The remaining costs were for session materials, printing/photocopying and refreshments. The average cost per CST participant per complete course was 269,265 TZS (101 US Dollars).

The CST training took consisted of two four-hour sessions and was attended by seven participants. The training was led by the master trainer for CST in Tanzania and supported by a research nurse. The total cost of the training was 282,500 TZS (106 US Dollars), of which 61% were staff costs. The remaining costs were for demonstration materials, printing/photocopying and refreshments. The average cost per training participant was 40,357 TZH (15 US Dollars).

#### Outcome Measures

Researchers completed pre-intervention outcomes with 41/49 participants (83.7%) and post-intervention outcomes with 40/49 participants (81.6%). Complete data for the outcomes was available for 36/49 participants (73.5%). Completing each assessment took approximately one hour per participant. For a group of eight participants, the total time spent on pre-and-post assessments was up to 16 h. Nine staff members were trained to support data collection. Despite training, some data were missing due to administrative errors.

Participants found the CSRI and RUD-Lite unacceptable due to the length and nature of the questions, particularly regarding information about their financial circumstances. No participants from the first four CST groups had complete data for the CSRI and RUD-Lite measures. Consequently, these were removed from the list of outcome measures.

#### Qualitative Data Post-CST Interviews

Following the CST groups, 31 interviews took place. This comprised 14 people with dementia and 12 carers from the first two CST groups, whose characteristics are outlined in Table 3. Five healthcare professionals (two occupational therapists, one nurse, and two psychiatrists) took part in interviews at the end of the CST programme.

**Table 3:** Demographic information of qualitative interview participants

Characteristic	Person with dementia ^a^		Carer ^b^	
	*n*	%	*n*	%
Sex				
Female	5	35.7	8	66.7
Male	9	64.3	4	33.3
Tribe				
Chagga	12	85.7	-	-
Pare	1	7.1	-	-
Shambaa	1	7.1	-	-
Marital status				
Married	10	71.4	10	83.3
Divorced	1	7.1	1	8.3
Widowed	2	14.3	0	0
Single	1	7.1	1	8.3
Primary language				
Kiswahili	13	92.9	-	-
Kichagga	1	7.1	-	-
Religion				
Christian	13	92.9	-	-
Muslim	1	7.1	-	-
Education level				
Completed Primary	6	42.3	5	43.9
Completed Secondary	5	35.7	4	36.6
Completed Tertiary	1	7.1	3	19.5
None	2	14.3	0	0
Relationship to person with dementia				
Son/Daughter	-	-	2	16.7
Spouse	-	-	6	50
Sibling	-	-	3	19.5
Another relative	-	-	1	8.3

Note. *N* = 26 (^a^*n* = 14; ^b^*n* = 12). People with dementia were on average 72.6 years old (SD = 9.0 years) and carers were on average 61.6 years (SD = 10.1 years)

Interviewees reflected on the perceived challenges and successes of the CST programme and gave recommendations for future practice. Qualitative findings are presented by CFIR domain: (1) Innovation characteristics; (2) Outer setting; (3) Inner setting; (4) Characteristics of individuals; and (5) Implementation processes. Table 4 summarises all relevant CFIR domains, constructs and context-specific descriptions. Within the text, CFIR domains are presented in parentheses.

**Table 4:** CFIR domains and constructs identified in qualitative interviews with setting-specific description

CFIR domain and construct	Description of construct within this setting	Barrier or facilitator
Innovation		
Innovation Relative Advantage	Carer perceived positive changes in cognition, social functioning and wellbeing of people with dementia.	Facilitator
Innovation Adaptability	Cultural adaptability of CST. Opportunities for further adaptation.	Facilitator
Outer setting		
Critical Incidents	COVID pandemic and its impact on the CST sessions.	Barrier
Local Attitudes	Need for improved dementia awareness. Misconceptions about the cause of dementia.	Barrier
Inner setting		
Structural Characteristics; Work Infrastructure	Staff were available and given protected time to deliver CST during this study.	Facilitator
Relational Connections	Interdisciplinary approach: occupational therapy, psychiatry, nursing and outpatient departments involved.	Facilitator
Culture; Learning-Centeredness	Staff gained knowledge and skills through CST programme delivery.	Facilitator
Compatibility	Fit of the intervention into diagnosis and referral pathways and existing service delivery. Group psychotherapies are delivered at the hospital.	Facilitator
Available Resources; Funding	Participants’ transport costs are reimbursed.	Facilitator
Available Resources; Space	Lack of suitable available space to deliver CST.	Barrier
Access to knowledge and information	Group leaders received training, or had previous experience of delivering CST.	Facilitator
Individuals		
High-Level Leaders	Need for higher-order leaders’ support.	Barrier
Other Implementation Support	Need for a carer to support and attend sessions, but presence can be distracting.	Both
Individuals (cont.)		
Other Implementation Support (cont.)	Improved dementia awareness/ understanding reported by carers.	Facilitator
Innovation Deliverers	Group leaders had previous experience of delivering CST.	Facilitator
	Professional development, supporting patients.	Facilitator
Innovation Recipients	People with dementia have a lack of stimulation in the home environment.	Facilitator
	Cost and complexity of transport to attend.	Barrier
	Motivated to improve symptoms, but some false expectations of recovery.	Both
Implementation process		
Tailoring Strategies	Two sessions run on the same day to reduce transport costs and burden.	Facilitator
Engaging; Innovation Recipients	Recruitment from outpatients was deemed more effective than from psychiatry, as patients were in the mild stages of dementia.	Facilitator
Reflecting & Evaluating; Innovation	Pre and post-assessments were time-consuming.	Barrier
Adapting	A desire for longer-term CST. Need for regional provision and recruitment. Need for advertising (Recommendations for future)	Facilitator

#### Innovation Characteristics

The following characteristics of CST were perceived to support delivery in the study setting. Overall, the intervention was perceived as acceptable. Almost all people with dementia or their carers reported perceived benefits, including improved wellbeing or mood, increased social functioning and improved cognition (CFIR construct: Innovation Relative Advantage).

“I would say this thing has made me happy because himself, he is very happy, he has liked it, and that is why he has been attending all sessions. It makes him laugh… and meeting different people.” Carer 5.

“It has helped me a lot. I have met many people and it has helped me in recalling.” Participant 10.

Some carers reported that the person with dementia was more active and engaged at home and in their day-to-day activities compared to before the CST groups. However, others felt their level of occupation had not changed.

“Until now watering the flowers was something that he was not doing, but now he takes his gallon and waters the flowers. Then he can sing by himself, songs that he knows.” Carer 6.

Two healthcare professionals remarked on the adaptability of CST, in particular, that CST had been culturally adapted successfully. One suggested that it could be adapted further to focus on activities of daily living or activities that were important to people with dementia and their carers (CFIR construct: Innovation Adaptability).

“I think they should also look at more cultural-based activities or activities which are like their day-to-day activities.” Healthcare professional 4.

#### Outer Setting

The following barriers to intervention delivery related to the outer setting (in this instance, the community and country context). The majority of interviewees highlighted the need for improved dementia awareness in the general public, which was also a key point in the phase 1 stakeholder meetings (CFIR construct: Local Attitudes).

“My request is that this should be known as a problem just like other problems. To the whole community and the whole country. Because this is a thing that can develop, many people are losing memories and developing dementia, the elders are having dementia. So you can give out leaflets that explain this condition.” Carer 3.

To address this barrier, a dementia awareness course was developed and delivered. Group leaders also educated carers about dementia and CST as part of the recruitment process.

“They become aware [of] the CST, through giving them information and explaining to them…That these clients have dementia, then we explained to them we were doing the CST. What is it about? How can it help those people?” Healthcare professional 2.

There were misconceptions about what had caused the dementia, including witchcraft or a loss or change of religion. Others felt that it was a normal part of getting older, and some attributed dementia to stress or other health conditions such as diabetes. Many carers hinted at feelings of stigma or embarrassment, as shown by a reluctance to disclose the diagnosis to others. Around half of the caregiver pairs had not shared with others that they were attending CST.

“I prefer those of my home and my family to know [rather] than those neighbouring to avoid misconceptions. It’s always good to focus on your family and the problems they go through.” Carer 7.

Additionally, the impact of COVID-19 meant that groups were delayed, and social distancing and mask-wearing guidelines were in place (CFIR construct: Critical Incidents).

“It affected [us] because we were in a training that involved [a] gathering of individuals. Even the seating arrangement and guidelines to prevent it had to be followed… Everyone was worried in every gathering that we attended. We were wearing name tags with names. If you are far from the person you could not easily tell who was speaking.” Participant 7.

#### Inner Setting

The majority of barriers and facilitators are related to the hospital setting. Staff were given protected time to deliver CST during the study. However, most healthcare professionals questioned what would happen at the end of the funding period. They highlighted a need for permanent staff in a dementia clinic (CFIR construct: Structural Characteristics, Work Infrastructure).

“I think that would be the next big thing. Not just for CST, but in general, to have a solid department caring for people with dementia in general, I think having an occupational therapist is something very important. It does feel like if the study stops, some things will also kind of come to a halt.” Healthcare professional 5.

Many people noted the lack of an appropriate area for conducting CST sessions and providing support to patients with dementia (CFIR construct: Available Resources, Space).

“It was a room that more than one group was depending on. This disease should have a special department with its ward, its place and toilets, so there should not be any disturbance. For example, there was a day when there was a meeting there in that room, we waited until 12 pm. 11 am is when we started, so there is a jam.” Participant 13.

“To have a special place, special day for them, we have a special team to give them their needs.” Healthcare professional 5.

During the study period, participants were reimbursed for their transportation costs, and CST was provided to them free of charge. Almost all participants found this support helpful. However, some reported that they did not always have the money upfront to cover their transport costs since they had to travel to the hospital to get reimbursed for each session. Some participants said they could not afford to attend the sessions if the costs were not reimbursed (CFIR construct: Available Resources, Funding).

“It depends if I get the fare, as you can see nowadays money is hard to get.” Participant 14.

“It would depend, if I get transport I [could] come. You prepare yourself because it is your problem and you have to struggle to solve it.” Participant 7.

All staff reported that CST was compatible with existing diagnosis and referral pathways and service delivery (CFIR construct: Compatibility). The delivery of CST involved an interdisciplinary approach involving occupational therapy, psychiatry, nursing, and outpatient departments, which was considered beneficial from a task-shifting perspective (CFIR construct: Relational Connections).

“It fits in really well with our clinic schedule, it fits in really well [with] the environment. It doesn’t really push people beyond their normal responsibilities too much. I think potentially, as our department is growing, we are expecting to have an occupational therapist trained in mental health. We have nurses who have already been observing, and they’re aware of what the intervention looks like. They also seem quite invested. It is something that could be implemented in the future.” Healthcare professional 5.

“To involve other healthcare workers, apart from just the occupational therapist, maybe to even involve the mental health nurse, or even just nurses, who are in the department here in psychiatry, or even medical doctors… because we are short staffed. So, if the knowledge can be passed to other healthcare providers, it can be easy to administer there.” Healthcare professional 4.

Staff had received CST training or had previous experience delivering CST (CFIR construct: Access to Knowledge and Information). Additionally, staff felt they gained knowledge and skills from the CST programme delivery (CFIR construct: Culture, Learning-Centeredness).

#### Characteristics of Individuals

This section presents the key attributes of the individuals involved in the implementation process, including hospital and government leaders, CST group leaders, people with dementia, and caregivers. These relate to the COM-B model (Michie et al., 2011).

The need for support from hospital leadership was raised, as well as the need for the government to drive the agenda for dementia awareness and support (CFIR construct: High-Level Leaders, Motivation).

“We started of course via this project, but I think even in the absence to continue. But what can be done? If the hospital administrators, those leaders of the hospital institution can take this into their consideration, this can be giving an impact, positive impact, in our institution.” Healthcare professional 1.

Many group leaders had previous experience delivering CST, which supported implementation. They were motivated to participate in the project for personal and professional development and to help patients (CFIR construct: Innovation Deliverers, Motivation).

“We feel proud. We do something potential in the community. Sometimes people are visiting us to see what are we doing here. So, for us, it is something unique which was not there before.” Healthcare professional 1.

Many people with dementia were motivated to participate in CST because they believed it would improve their memory, and many felt that their cognition had improved. However, many misunderstood that CST had ‘cured’ their memory problems. Describing CST as a treatment to support their symptoms may have been misinterpreted as an absolute cure (CFIR construct: Innovation Recipients, Motivation).

“People should not reach a point that they have complete dementia. It is a very difficult thing. It is a bad thing. People should check their health, especially cognition health, at the hospitals. As the way we came and stayed and got advice about what to do and later you find yourself recovering. The cognition health recovers and moves on with life.” Participant 2.

“If I saw the symptoms were starting again I would immediately run to the course, I mean faster. But I believe I have recovered.” Participant 5.

Many carers reported an improved understanding of dementia. Some remarked that they now realised that the frustrations they felt towards the person with dementia were caused by their symptoms. As a result, they were able to be more patient and understanding towards the person with dementia. Others reported encouraging the person with dementia to stay active and engage in day-to-day activities at home (CFIR construct: Other Implementation Support, Capability).

“I used to do these things for him but lately I have told him to practise it by himself doing those activities.” Carer 4.

#### Implementation Processes

Instead of conducting sessions on separate days, both sessions were held on the same day to reduce transport costs and burden (CFIR construct: Tailoring Strategies). Two patient engagement and recruitment approaches were used: screening outpatients for dementia and direct referrals of people with dementia from the psychiatry caseload. Two healthcare professionals reported that screening was the most suitable strategy as it identified people with dementia in the earlier stages and also raised awareness of dementia in other hospital specialities (CFIR construct: Engaging, Innovation Recipients).

“I think the screening first. Because I think at the screening stage, that’s when you get, you can get patients who are not in the severe form. So, once you get them about the mild and moderate, it can be it will be good for intervention. Most of the time, we get [referrals] from the other departments or other hospitals, they come in [the] severe state.” Healthcare professional 5.

“The more preferable option for implementation purposes, the screening would be more preferable, just because then it’s not someone from psychiatry doing all of the referrals, but it’s other people being more aware and integrating screening into other clinics.” Healthcare professional 4.

Pre- and post-assessments were reported as time-consuming, and some of the outcome measures were not acceptable to participants, particularly those related to personal economic circumstances (CFIR construct: Reflecting and Evaluating, Innovation).

“For those who are okay with their studies and active, it’s a case of 45 [minutes] to one hour. If they go direct to the points. But there are those who are story makers (laughs) you must take one and a half hours, but they bring a lot of stories.” Healthcare professional 3.

Finally, both groups of stakeholders made recommendations for future CST delivery. Nearly all people with dementia reported a desire for the CST sessions to continue (CFIR construct: Adapting).

“The course should continue. When he comes here and meets others maybe he will be different, so it should be prolonged to them and to others.” Carer 13.

“It is just to insist that people should continue to get exercises. It should be prolonged for the people with such problems and this will reduce the stress a lot.” Carer 6.

Carers and people with dementia also expressed the need for local support services within their communities. They emphasised the need for advertising and engagement with local leaders to identify people with dementia. This approach was seen as a way to address challenges related to distance, cost and complexity of travel to the regional hospital.

“I would like other people also to get this service because nationwide, there are many [people with dementia]. [I] think it is a good service so should be extended until in the villages.” Participant 13.

## Discussion

This multi-phase project investigated the perceived barriers and facilitators to implementing CST for dementia in Tanzania. We consulted key stakeholders to create an implementation plan of 20 strategies related to 12 CFIR constructs. We then explored the feasibility and acceptability of CST implementation and the success of implementation strategies in a study of CST in a hospital setting. From qualitative interviews with study participants and staff involved in implementation, we identified 18 CFIR constructs considered barriers or facilitators to implementing CST. By describing these factors according to a widely used determinant implementation framework, this work can guide and inform future implementation of CST in low-resource settings. This project is part of a growing field of work translating research evidence into practice and policy in Africa. (Farombi et al., 2023; Ojagbemi & Daley, 2015), as recommended by The Africa Dementia Consortium (Akinyemi et al., 2022).

CST had already been successfully culturally adapted from the protocol developed in the UK for use in Tanzania (Mkenda et al., 2018). However, cultural adaptation alone does not guarantee successful implementation. It is important to assess intervention implementation in the new clinical and cultural context and tailor implementation strategies accordingly, rather than relying on a ‘one-size fits all’ approach (Bauman et al., 2017; Powell et al., 2017). Our work highlights the importance of exploring the implementation of evidence-based interventions in real-world settings and the importance of stakeholder consultation and engagement within this process.

Several implementation strategies included in the phase 2 plan were not delivered or perceived as important within the phase 3 study of CST. For instance, initial stakeholder engagement highlighted the importance of working with media outlets to promote CST and contacting insurance companies about covering CST. Neither of these was carried out in the phase 3 study, but these could be important strategies in a larger-scale implementation project. The phase 3 study of CST revealed barriers and facilitators that had not been considered in the earlier phases, such as the need for a dedicated space for CST sessions for people with dementia and the use of various recruitment and referral pathways. This shows a disconnect between the perceived importance of these strategies and the reality of their implementation, as well as the importance of testing and evaluating implementation strategies.

### Referral Pathways

Eight of the 49 participants were directly recruited from the psychiatry patient caseload. The majority of people with dementia on the caseload are in the later stages of the condition and, therefore, not eligible for CST. Screening of outpatients helped to identify patients with mild-to-moderate dementia, but many who screened positive for potential dementia could not be contacted, possibly due to stigma or low levels of dementia awareness. This emphasises the need for a longer-term program of awareness raising and screening, which could aid earlier diagnosis.

### CST Trainers

Before this study, there was only one CST trainer in Tanzania. Now, there are three more trainers, which allows for CST training to be delivered to group facilitators, aiding in the decentralisation of the training from the International CST Centre at University College London.

### Sustainability

Almost all participants expressed a desire for longer-term follow-up, and many wished for groups to continue beyond the initial seven weeks. This need could be met through implementing a 24-week ‘Maintenance CST’ programme, which has demonstrated positive impacts on quality of life and activities of daily living at a 6-month follow-up (Orrell et al., 2014). Healthcare professionals were concerned that activities might stop at the end of the project funding period and reported the need for a specialist department and space dedicated to caring for people with dementia, which could incorporate a range of support services and interventions.

### Location and Transport

Attendance at CST sessions was high (81%), despite long travel distances. However, transport costs were reimbursed, and many participants reported they could not attend if funding was not available. Stakeholders had initially suggested that CST sessions should take place in community settings. However, a decision was made to run sessions at the hospital due to screening and recruitment taking place there. Previous trials of CST and dementia prevalence studies have successfully employed community screening (Paddick et al., 2017b; Yoseph et al., 2021), so future work could explore recruitment and referral pathways for sustainable provision in rural areas.

### Measurement Burden

Pre- and post-intervention outcome measures were collected. A large measurement burden existed, especially compared to the efficiency of delivering a group intervention. Assessments took approximately one hour per participant, totalling up to 16 h per CST group. This is noticeably high, considering a CST course runs for 14 h. Nine staff members were trained to conduct assessments compared to four CST group leaders. If used in routine practice, short and culturally validated measures of cognition and quality of life for the person with dementia are recommended, as these are the primary outcomes of CST (Woods et al., 2023).

### Task-Shifting

The interdisciplinary approach employed in this study lends itself to a task-shifting approach, especially because CST does not need to be delivered by specialists. Task-shifting approaches have been presented as solutions to the lack of specialist staff and limited resources supporting people with dementia. Healthcare that is usually delivered by specialists is moved to non-specialist community teams and primary care under the training and supervision of specialist teams, resulting in cost savings and improved patient outcomes (Joshi et al., 2014; Prince et al., 2016). However, it is important that the task shifting is formalised, roles and responsibilities are made clear, and specialists provide appropriate training and supervision. A study of task-shifting in dementia care pathways in South Africa faced barriers, including limited resources and a lack of staff training and awareness (Prince et al., 2016). Additionally, a study of task-shifting in the pharmaceutical industry in Tanzania showed that it was employed as a coping mechanism in response to a lack of staff rather than being a formal and planned strategy (Wiedenmayer et al., 2015).

### Limitations

The study sample may not fully represent the general population of people with dementia and their caregivers. For instance, 60% of participants had completed secondary education, almost double the current secondary school enrolment rate (US AID, 2024). The study was carried out at a large university hospital in an urban area, which might have led to a self-selecting sample of individuals with higher education and greater affluence, who were more likely to have the means to attend appointments and CST sessions.

There was a high rate of non-participation. Only 41.6% who screened positive for suspected dementia were contactable to organise a follow-up diagnostic appointment. The rate of non-attendance at these follow-up appointments was 43.1%. Due to limited resources, we were unable to follow up with those who did not engage so were not able to explore reasons for non-attendance. It is possible that our results are biased towards individuals more likely to attend CST groups and, therefore, give a more positive view of the programme.

Transport costs were reimbursed for the study, and many participants reported they would not have been able to attend if this funding was not available. However, due to limited funding, it is unlikely that transport costs would be reimbursed in routine care. Therefore, the attendance rates in this study may not reflect the expected rates for regular care.

A key element of evaluating implementation is assessing fidelity to the intervention (Proctor et al., 2011). We did not carry out a formal assessment of fidelity. However, a sample of the CST groups was observed by the master trainer and research programme manager.

Interviews with healthcare professionals in phase 3 were conducted in English by a UK-based researcher who was not a member of the immediate research team. This was to limit response bias and encourage honest and critical feedback. However, the interview was limited to people who spoke English, and it may have compromised the representation of non-English speakers. However, this likely had a minimal effect as healthcare professionals in Tanzania are taught in English.

Finally, the CFIR was updated part way through this research project. The interview guides were developed based on the original framework (Damschroder et al., 2009), but we analysed the qualitative interviews using the updated framework (Damschroder et al., 2022). This decision was made because the updated constructs better capture the experiences of intervention recipients and are more suitable for use in low-resource settings (Damschroder et al., 2022). Additionally, the revised framework can be mapped back to the original, so this has had a negligible effect on the qualitative results.

### Future Directions

A sustainable funding plan and a dementia-friendly space within the hospital are needed. Additionally, since transport costs are a significant barrier to attendance, future CST programmes could be delivered in the community. Future work could explore how CST can fit into a wider dementia awareness and early diagnosis programme, and post-diagnostic support. Finally, the recommendations for implementation could be further explored in other sites and countries.

## Conclusion

Following extensive stakeholder engagement to develop and test implementation plans, we found that CST was compatible with the standards of care in a tertiary referral hospital in northern Tanzania. Participants and staff expressed a desire for the CST groups and support for people with dementia to continue. Using the CFIR allowed us to identify barriers and facilitators to successful implementation, which can be further explored in other settings and contexts. Key facilitators to CST implementation included staff training and support, funding arrangements, referral pathways such as existing patient caseloads or screening programmes, and treatment pathways. The results of this study may guide future efforts to implement CST and other psychosocial interventions for dementia in low-resource settings.

## Electronic Supplementary Material

Below is the link to the electronic supplementary material.

Supplementary Material 1

Supplementary Material 2

Supplementary Material 3

Supplementary Material 4

Supplementary Material 5
