# Supplementary material for: Implementing Cognitive Stimulation Therapy (CST) for Dementia in a Low-Resource Setting: A Case Study in Tanzania Exploring Barriers, Facilitators, and Recommendations for Practice
Source: Glob Implement Res Appl. 2025 Jan 11;5(1):106–23. doi: 10.1007/s43477-024-00142-6 (PMC11821707; doi:10.1007/s43477-024-00142-6)
Supplement: Supplementary file 2 — Supplementary Material 2 [file 43477_2024_142_MOESM2_ESM.docx]

# Supplementary file 1

**Local Checklist**

**Below are some commonly encountered problems when running CST groups. This checklist should be completed by a CST facilitator prior to running their first group.**

**Location of Group (building and region)**

___________________________________________

**Participants**

Do all of your participants meet the ICD-11 criteria for dementia?

**Facilities**

Will the session be held in a private place where participants will not be interrupted?

Is the CST session in a neutral setting (e.g. it is not in a church)?

Will there be access to drinking water?

Is there a toilet nearby?

Is there a table that can be used?

Are there enough chairs?

Are the chairs the right height?

**COVID-19 safety**

Does the room have ventilation? i.e. windows can be opened to allow air flow

Are masks available for participants and facilitators to wear?

Are handwashing facilities or hand sanitizer available?

Are chairs set up to allow social distancing?

**Travel and Timing**

Can the building be reached by public transport?

Is the building close enough to people’s homes?

Have you checked when the local market day is?

Have you agreed which times and dates the CST sessions will be with carers?

Have you considered whether rainy season will affect attendance?

**Materials**

Have you got all the materials you need (e.g. local foods for the food session)?

Is there a contingency plan for sessions where you will need electricity?

Do you have glasses for participants?
