# Supplementary material for: Implementing Cognitive Stimulation Therapy (CST) for Dementia in a Low-Resource Setting: A Case Study in Tanzania Exploring Barriers, Facilitators, and Recommendations for Practice
Source: Glob Implement Res Appl. 2025 Jan 11;5(1):106–23. doi: 10.1007/s43477-024-00142-6 (PMC11821707; doi:10.1007/s43477-024-00142-6)
Supplement: Supplementary file 3 — Supplementary Material 3 [file 43477_2024_142_MOESM3_ESM.docx]

# Supplementary file 2

**Guidance for CST facilitators when running groups**

1. Make sure the chairs are the right height for the table and remove any chairs that are not being used.
2. Seat all participants in a circle around the same table.
3. Make sure that COVID safety measures are in place. If indoors, open the windows to allow ventilation. Masks should be worn and participants and facilitators should socially distance from each other. Regular handwashing/ sanitising should take place and objects and surfaces should be sanitised.
4. Facilitators should sit at the table with the participants rather than stand.
5. Facilitators should not wear clinical coats or uniforms during sessions.
6. Avoid distractions where possible. Do not bring food or drinks to the group unless it is part of a session.
7. There should only be two facilitators and they should be spread evenly amongst the group. If there is a participant who cannot hear well or is not joining in, a facilitator can sit next to this person and gently encourage them to take part.
8. Facilitators should always be concentrating on the group. No phones at all and no note taking unless it is absolutely necessary.
9. Have conversations with your participants and find out more about them e.g. if a song is playing, ask them what they like about it.
10. Most importantly, get to know the participants and have fun together!
