# Supplementary material for: Implementing Cognitive Stimulation Therapy (CST) for Dementia in a Low-Resource Setting: A Case Study in Tanzania Exploring Barriers, Facilitators, and Recommendations for Practice
Source: Glob Implement Res Appl. 2025 Jan 11;5(1):106–23. doi: 10.1007/s43477-024-00142-6 (PMC11821707; doi:10.1007/s43477-024-00142-6)
Supplement: Supplementary file 4 — Supplementary Material 4 [file 43477_2024_142_MOESM4_ESM.docx]

**CST-International Interview Guide for Group 2:**

**Healthcare professionals and CST group leaders / facilitators**

Table of Contents

[Introduction and background 3](#_Toc33094880)

[*Researcher and Research Team 3*](#_Toc33094881)

[*CST-International 3*](#_Toc33094882)

[*Purpose of the Interview 3*](#_Toc33094883)

[*Audio Recording 4*](#_Toc33094884)

[*Pre-interview questions 4*](#_Toc33094885)

[Demographic Information 5](#_Toc33094886)

[Open-ended Description of Implementation 6](#_Toc33094887)

[Pre-Implementation Experiences 9](#_Toc33094888)

[Implementation Experiences 11](#_Toc33094889)

[Operational Experiences 12](#_Toc33094890)

[Patient Experience 13](#_Toc33094891)

[Costs 14](#_Toc33094892)

[Overall Experience and Recommendations 15](#_Toc33094893)

[Final Question 15](#_Toc33094894)

[End of Interview 16](#_Toc33094895)

Introduction and background

The following script should be used for all Group 2 participants who take part in qualitative interviews. Please note that this is guide is not intended to be read out verbatim except for close-ended questions. The purpose of the guide is to ensure that all topics of interest are covered. Open narrations of participants’ experiences is encouraged and researchers should probe for more details where they feel it is appropriate.

Relevant Consolidated Framework for Implementation Research (CFIR) constructs are included below each question. Constructs are included from both the original framework (Damschroder et al., 2009) and the updated framework (Damschroder et al., 2022).

Researcher and Research Team

Hello, my name is [name of interviewer]. *Introduce self and other team members in the room.*

CST-International

The purpose of CST-International is to better understand the barriers and facilitators for implementing Cognitive Stimulation Therapy for people with dementia in Tanzania. Our aim to improve access to CST so that in future, more people with dementia can take part. We want to take this time to talk to you about your experience so far with the CST-International programme. Your thoughts will help us evaluate how CST has been implemented and variation between regions and countries. We want to understand the challenges and successes of adopting and implementing CST so that we can maximise the likelihood that other sites will be successful.

Purpose of the Interview

Your interview will help us to better understand the challenges and successes of CST at your facility. We will be interviewing multiple people at your facility to gain multiple perspectives. There are no right or wrong answers. We are really interested in learning more about your own experience with this programme.

We are going to ask you questions about your experience facilitating CST for your facility.

Audio Recording

This interview will be audio taped so that we have an accurate record of your thoughts. Please be assured that the tapes and your transcript will be kept confidential. Leadership at your facility and any co-workers will not have access to your responses. The only exception to this is if you tell me information that I have a duty to report. This would be reporting harm to yourself or others, criminal activity or professional misconduct. If you tell me something that I have a duty to report, I will discuss this with you first. Once your interview has been transcribed, the audio recording will be destroyed.

To help ensure confidentiality, it would help if you don’t mention the name of your facility or hospital wherever possible and also don’t mention names of specific staff members of service users during the interview. If, at any point, you feel the questions are too sensitive or you would prefer not to answer a question, you do not have to answer. The interview is voluntary, which means you can stop at any time and you do not have to give a reason for stopping.

Pre-interview questions

Do you have any questions for me?

Are you ready to begin? I will start the recording now.

Demographic Information

We will now begin the main part of the interview. We want to hear your thoughts so please do not hesitate to share whatever you believe might be related to any of the topics. Now I would like to ask you a few questions to help me understand your role and who was involved with CST at your site.

1. **Will you please describe your role at the facility/ hospital/ service?**

*Probes:*

- *What is your title and role within your organisation?*
- *Who do you report to?*
- *Tell me about your role for CST?*

1. **Do you work with other clinicians outside this role?**

*Probes:*

- *Societies? Conferences? Regional meetings?*
- *Do you attend professional or work-related conferences or outside training?*
- *How have your network contacts influenced your work?*

*CFIR Constructs:*

*Cosmopolitanism (2009) / Partnerships & Connections (2022)*

1. **Who else did you work with to deliver CST?**

*Probes:*

- *Doctors, nurses, physiotherapists, occupational therapists, admin?*

*CFIR Constructs:*

*Networks and communications (2009) / Relational Connections and Communications (2022)*

Open-ended Description of Implementation

Could you please describe in detail the process by which CST was set up in your experience?

*Probes:*

- *Use any background information that might be available in terms of milestones (date of training, date of first CST group)*
- *How are participants recruited? Who recruits them?*
- *Who schedules the CST groups and how is this related to the attendees and their family?*
- *Who monitors the progress of attendees and how do they monitor this?*
- *Do you have all the materials needed for CST (local food/ access to music etc)?*
- *How many CST groups have you been a part of? Are there future groups planned?*
- *Are you aware of any marketing or adverts about CST?*
- *What do carers or family members do when the CST group is running?*
- *What was your experience of delivering the pre/post measures?*

*CFIR Constructs:*

*Planning (2009 / 2022)*

*Tailoring strategies (2022)*

*Executing (2009) / Doing (2022)*

*Reflecting & Evaluating (2009) / Reflecting & Evaluating; Implementation/Innovation (2022)*

*Available Resources (2009) / Available Resources; Materials & Equipment (2022)*

*Engaging (2009) / Engaging; Innovation Recipients (2022)*

*Access to knowledge and information (2009 / 2022)*

Pre-Implementation Experiences

I would like to hear more about how the CST initially started at your site and how you and others viewed it

1. **How did your site become involved with this intervention? How did you personally get involved?**

*Probes:*

- *Would you say the implementation of CST was more externally driven by others or more internally motivated by the team at your facility? Why?*
- *Did someone at your site or you have a say in whether you participated?*
- *Did you feel like your site’s participation was voluntary?*

*CFIR Constructs:*

*Innovation Deliverers; Capability/Opportunity/Motivation/Need (2022)*

*Engaging (2009) / Engaging; Innovation Deliverers (2022)*

1. **Did you see a need for this type of intervention? Why or why not?**

*Probes:*

- *At that time, how did you think implementing CST would help or meet the needs of clinicians? To what extent did it actually help?*
- *Improvement in work process (amount or type)?*
- *How did you think CST would help or meet the needs of patients? To what extent did it actually meet that need?*
- *Better patient outcomes? Which outcomes?*

*CFIR Constructs:*

*Relative Advantage (2009) / Innovation Relative Advantage (2022)*

*Compatibility (2009 / 2022)*

*Patient Needs and Resources (2009) / Innovation Recipients; Capability/Opportunity/Motivation/Need (2022)*

1. **What kind of services were you already offering to people who have dementia or memory problems at your site?**

*Probes:*

- *To what extent were the existing services multi-disciplinary?*
- *How were people with dementia being treated?*
- *To what extent?*
- *Were there other similar initiatives?*
- *Do you perceive CST as being a superior alternative? Why or why not?*
- *To what degree was there “competition” for funds, time, or attention because of other initiatives that may have been taking place concurrently?*

*CFIR Constructs:*

*Relative Advantage (2009) / Innovation Relative Advantage (2022)*

*Relative Priority (2009 / 2022)*

*Compatibility (2009 / 2022)*

*Available Resources (2009) / Available Resources; Funding (2022)*

Implementation Experiences

Now, I’d like to ask some questions about your experience getting CST implemented and the type and level of support you had.

1. **On a scale of 0-10, with 0 being very easy and 10 being nearly impossible, how difficult was CST to implement in your facility? Why?**

*Probes:*

- *What were the barriers you experienced in implementing each of the components (identifying meeting space, identifying people with dementia, protected time to run groups etc)?*
- *What were the facilitators you experienced in implementing each of the components?*

*CFIR Constructs:*

*Complexity (2009 / 2022)*

**The next questions relate to Operational Experiences:**

1. **Did you experience any problems in running CST? Why?**

*Prompts:*

- *Equipment*
- *Venue/space*
- *Participants*
  - *Cultural differences*
  - *Gender*
  - *Language barriers*
  - *Dementia severity*

*CFIR Constructs:*

*Available Resources (2009) / Available Resources; Funding/Space/Materials & Equipment (2022)*

*Assessing Needs; Innovation Recipients (2022)*

*Assessing Context*

**2) Do you have any suggestions for avoiding these problems?**

**The next questions relate to Patient Experience:**

1. **In your opinion, why did people with dementia participate in CST?**

*Probes:*

- *Appeal of CST groups*
- *Appeal of meeting others like them*
- *Engagement with hospital services*
- *Carer respite*
- *What do you think they hoped to gain by attending CST groups?*

*CFIR Constructs:*

*Patient Needs and Resources (2009) / Innovation Recipients; Capability/Opportunity/Motivation/Need (2022)*

*Compatibility (2009 / 2022)*

1. **What did people with dementia think about CST?**

*Probes:*

- *What impact did CST have on participants (cognition, wellbeing, opportunity for social engagement, carer wellbeing etc)*
- *What did people with dementia expect from CST*
- *Were there any participant characteristics that impacted the group (e.g. gender/religion/ language)?*

*CFIR Constructs:*

*Patient Needs and Resources (2009) / Innovation Recipients; Capability/Opportunity/Motivation/Need (2022)*

*Relative Advantage (2009) / Innovation Relative Advantage (2022)*

The next questions are about Costs:

1. **What kind of support did you receive for participating in CST?**

*Probes:*

- *Protected time for scheduling/ running group*
- *Relief from other duties*
- *Assistance from other staff*
- *Funds to buy equipment*
- *Were there resources or support that would have helped you implement CST that you did not receive?*

*CFIR Constructs:*

*Available Resources (2009) / Available Resources; Funding/Space/Materials & Equipment (2022)*

*Structural Characteristics (2009) / Structural Characteristics; Work Infrastructure (2022)*

1. **Did you have any funding constraints whilst attempting to implement CST?**

*Probes:*

- *Inadequate staffing*
- *Staff turnover*
- *Inadequate time*
- *Competing priorities*

*CFIR Constructs:*

*Available Resources (2009) / Available Resources; Funding (2022)*

*Structural Characteristics (2009) / Structural Characteristics; Work Infrastructure (2022)*

*Relative Priority (2009 / 2022)*

Overall Experience and Recommendations

1. **Generally speaking, what do your colleagues at your facility think of the CST programme?**
2. **On a scale of 0 – 10, how successful do you think CST has been at your facility? Why? (NOTE: interview can define success in any way).**
3. **If you had the option, would you recommend continuing CST at your facility? Why/why not?**

*Probes:*

- *What is needed to keep CST going at your site?*
- *What type of justification would you need to show to keep this intervention going over the long term?*

1. **Would you recommend CST to other sites in this region/ country? Why/ why not?**
2. **Do you have any suggestions for other sites who have not yet started using CST?**
3. **How would you improve the CST programme? Why?**

Final Question

1. **Is there anything else you would like to tell us about your experience of CST at your facility?**

End of Interview

Thank you very much for answering these questions. I will now turn the recorder off. After we have analysed the results from all sites, we aim to submit our findings for publication in a peer-reviewed journal or we’ll present them at national and international conferences. If you would like any more information, please get in touch with the CST-International Programme Manager.

**END**

References

Damschroder, L. J., Aron, D. C., Keith, R. E., Kirsh, S. R., Alexander, J. A., & Lowery, J. C. (2009). Fostering implementation of health services research findings into practice: A consolidated framework for advancing implementation science. Implementation Science, 4(1). https://doi.org/10.1186/1748-5908-4-50

Damschroder, L. J., Reardon, C. M., Widerquist, M. A. O., & Lowery, J. (2022). The updated Consolidated Framework for Implementation Research based on user feedback. Implementation Science 2022 17:1, 17(1), 1–16. https://doi.org/10.1186/S13012-022-01245-0
