# Supplementary material for: Implementing Cognitive Stimulation Therapy (CST) for Dementia in a Low-Resource Setting: A Case Study in Tanzania Exploring Barriers, Facilitators, and Recommendations for Practice
Source: Glob Implement Res Appl. 2025 Jan 11;5(1):106–23. doi: 10.1007/s43477-024-00142-6 (PMC11821707; doi:10.1007/s43477-024-00142-6)
Supplement: Supplementary file 5 — Supplementary Material 5 [file 43477_2024_142_MOESM5_ESM.docx]

**CST-International Interview Guide for Group 3:**

**People with dementia**

Contents

[Introduction and Background 2](#_Toc172734920)

[Researcher and Research Team 2](#_Toc172734921)

[CST-International 2](#_Toc172734922)

[Purpose of the Interview 2](#_Toc172734923)

[Decision to take part 2](#_Toc172734924)

[Audio Recording 3](#_Toc172734925)

[Results of the study 3](#_Toc172734926)

[Pre-interview questions 3](#_Toc172734927)

[Demographic information 5](#_Toc172734928)

[Pre-implementation Experiences 6](#_Toc172734929)

[Patient Experience 7](#_Toc172734930)

[Impact of COVID-19 12](#_Toc172734931)

[Overall Experience and Recommendations 13](#_Toc172734932)

[Final Question 14](#_Toc172734933)

[End of Interview 14](#_Toc172734934)

# Introduction and Background

The following script should be used for all CST participants who take part in qualitative interviews. Please note that this guide is not intended to be read out verbatim except for close-ended questions. The purpose of the guide is to ensure that all topics of interest are covered. Open narration of participants' experiences is encouraged and researchers should probe for more details where they feel it is appropriate.

Relevant Consolidated Framework for Implementation Research (CFIR) constructs are included below each question. Constructs are included from both the original framework (Damschroder et al., 2009) and the updated framework (Damschroder et al., 2022).

## Researcher and Research Team

Hello, my name is [name of interviewer]. *Introduce self and other team members in the room.*

## CST-International

The purpose of CST-International is to understand how to implement Cognitive Stimulation Therapy for people with dementia in Tanzania. We aim to improve access to CST so that in future, more people with dementia can take part. We want to talk to you about your thoughts on the CST-International programme so far. Your thoughts will help us find out how well CST has worked and see the differences between regions and countries. We want to understand the problems and successes of using CST so that we can improve in the future.

## Purpose of the Interview

You have been invited to this interview to help us better understand the challenges and successes of CST. We will be interviewing people to see what they think. There are no right or wrong answers. We are really interested in learning more about your own experience with this programme. You have been chosen because you have attended a course of CST sessions.

## Decision to take part

It is up to you to decide whether to take part in this interview. If you decide to take part, you can ask to stop the interview at any time and do not need to give a reason for this. You can also decide not to take part at all.

## Audio Recording

This interview will be audio taped so that we have a record of your thoughts. The tapes and your transcript will be kept completely confidential. Friends and family will not have access to your responses. The only exception to this is if you tell me information that I must report. This would be anything that may lead to/ that describes harm to yourself or others, criminal activity or professional misconduct. If you tell me something that I have a duty to report, I will discuss this with you first. Once your interview has been written down, the audio recording will be destroyed.

To help ensure confidentiality, it would help if you don’t mention the name of your hospital if possible and don’t mention names of staff members or patients during the interview. If, at any point, you feel the questions are too sensitive or you would prefer not to answer a question, you do not have to answer. The interview is voluntary, which means you can stop at any time and you do not have to give a reason for stopping.

Do you have any questions for me?

We will now begin the main part of the interview. We want to hear your thoughts so please do not hesitate to share whatever you believe might be related to any of the topics

# Personal information

I would like to ask you a question to help me understand a bit about yourself.

1. **What kinds of memory problems did you experience before doing CST?**

*Probes:*

- *When did you first start noticing memory problems?*
- *How did your memory problems affect your day-to-day life?*
- *Do you interact with other people day-to-day? Can you give some examples?*
- *Had you heard of dementia before?*
- *Did you know you had dementia before attending CST?*
  - *If not, how was your experience being diagnosed at the hospital?*
- *How much do you know about dementia/ your condition?*
- *Did you experience any other problems relating to your dementia before CST?*

*CFIR Constructs:*

*Patient Needs and Resources (2009) / Innovation Recipients; Need / Capability (2022)*

1. **Do you see other healthcare workers for this or any other health conditions? If so, can you please describe these relationships?**

*Probes:*

- *Do you have any other diagnosed conditions?*
- *Do you attend any other therapy groups?*
- *Have these groups affected your involvement in CST?*
- *Have you ever tried anything like CST before?*

*CFIR Constructs:*

*Cosmopolitanism (2009) / Partnerships & Connections (2022)*

# Pre-implementation Experiences

1. **How did you hear about CST? What did you think about it? Why did you want to take part?**

*Probes:*

- *How were you invited to take part in CST?*
- *Was someone you know involved in the decision to take part?*
- *Did you feel like your participation was voluntary?*
- *What were you expecting? Were you provided with much information beforehand? Did it match these expectations?*
- *What did you think the aims were of CST?*

*CFIR Constructs:*

*Self-Efficacy / Innovation Recipients; Motivation/Capability (2022)*

*Intervention Source (2009) / Innovation Source (2022)*

*Knowledge and Beliefs about the Intervention (2009) / Innovation Recipients; Motivation (2022)*

1. **What did the facilitators/group leaders tell you about how CST worked?**

*Probes:*

- *Did you get any information from friends or family?*
- *Did the evidence influence your opinion of CST?*
- *Was CST a better alternative than other programmes that may have been available at that time?*

*CFIR Constructs:*

*Evidence Strength & Quality (2009) / Innovation Evidence-Base (2022)*

*Relative Advantage (2009) / Innovation Relative Advantage (2009)*

*Other Implementation Support; Motivation (2022)*

1. **What kind of services had already been offered to you?**

*Probes:*

- *How were you being treated for dementia?*
- *Have you been offered any medication for dementia?*
  - *If not, would you like to be able to access medication for dementia?*
  - *Would you rather have access to medication for dementia, or access to the CST groups?*
- *Do you think CST was better than the treatments you had already been offered? Why or why not?*

*CFIR Constructs:*

*Relative Advantage (2009) / Innovation Relative Advantage (2009)*

*Complexity (2009) / Innovation Complexity (2022)*

# Patient Experience

1. **In your opinion, why did you participate in CST?**

*Probes:*

- *Appeal:*
  - *What did you like?*
  - *What did you dislike?*
  - *What would you change?*
  - *Did you feel involved/ included?*
- *Facilitator:*
  - *Who ran your sessions?*
  - *Did the same person run your sessions?*
  - *What did you like or dislike about your facilitator/group leader?*
    - *Culture/language*
    - *Age*
    - *Gender*
  - *Did they seem engaged in the sessions?*
  - *Did they seem like they were confident in leading the sessions?*
  - *Did they seem well suited for the role?*
- *Other participants:*
  - *What did you think about them?*
  - *Did this affect the session? Why?*
  - *What did you think about the size of the group?*
  - *How engaged were the other participants in the group?*
- *Impact:*
  - *Have you noticed any changes in cognition/wellbeing/social engagement?*
  - *Have you noticed any changes in your family member/ friend who cares for you? Are your interactions with them similar to before or has this changed over the course of CST?*

*CFIR Constructs:*

*Patient Needs and Resources (2009) / Innovation Recipients; Capability/Opportunity/Motivation/Need (2022)*

*Tension for Change (2009 / 2022)*

*Compatibility (2009 / 2022)*

1. **How did you feel being in the group of people?**

*Probes:*

- *How did you find the sessions? How did they make you feel?*
- *How was it doing the sessions face-to-face?*
- *Did you mix/ interact with others during the group?*
  - *Yes/ no/ why not/ when? Examples?*
  - *Make any friends?*
  - *Did you feel involved/included?*
  - *Were you helped by others?*
  - *Did you trust others?*
  - *Did you feel respected by others?*
  - *Did you feel similar to others? How was talking to other people with dementia? How does this compare with day-to-day interactions?*
  - *What sort of things do you do with other people when not at the sessions?*
    - *Has this changed since coming to the sessions?*
  - *Were group members of different religious backgrounds/ tribes?*
    - *If so, did this cause any issues?*
    - *if not, why not?*
- *What did you talk about?*
  - *Did you have the chance to talk in the group?*
  - *If yes, how easy was it?*
  - *If not, why not?*
  - *Was it hard to say what you wanted to others?*
  - *Was there any sense of avoidance of important issues?*
  - *Did you ever feel afraid to share your feelings with others?*
  - How was the relationship between you and your group leader?

*CFIR Constructs:*

*Patient Needs and Resources (2009) Innovation Recipients; Capability/Opportunity/Motivation/Need (2022)*

*Relative Advantage (2009) / Innovation Relative Advantage (2009)*

1. **Can you tell me a bit about what activities you did in the CST sessions?**

*Probes:*

- *Which activities did you like/ not like? Why?*
- *Were the sessions what you were expecting? Did it match the information you were given prior to starting CST?*
  - *If not, what was different to what you were expecting?*
- *Did being in a group with others affect your experience of these activities?*
- *Did the sessions seem well organised?*

*CFIR Constructs:*

*Patient Needs and Resources (2009) Innovation Recipients; Capability/Opportunity/Motivation/Need (2022)*

*Relative Advantage (2009) / Innovation Relative Advantage (2009)*

*Available Resources (2009) / Available Resources; Materials & Equipment (2022)*

**4) Can you describe the place where CST takes place?**

*Probes:*

- *Was the day CST sessions were held on convenient for you?*
  - *If not, why not?*
  - *What would have made it more convenient for you?*
  - *For the sessions that were moved to a different day, did this cause much disruption?*
- *Was the place for CST convenient to get to?*
  - *If not, why not?*
  - *What would have made it more convenient for you?*
  - *Would you rather have CST delivered somewhere else?*
- *Where was your CST? How did you get there? Was it costly?*
- *How many were in your group?*
- *What language was CST delivered in?*
- *What did you think about the place where the CST sessions were held?*
  - *Was it what you were expecting?*
  - *Is there anything you did not like about the place where the CST sessions took place?*
  - *Did the place seem appropriate for the CST sessions?*
  - *What did you think of the sessions being held at the hospital?*

*CFIR Constructs:*

*Patient Needs and Resources (2009) Innovation Recipients; Capability/Opportunity/Motivation/Need (2022)*

*Available Resources (2009) / Available Resources; Funding/Space (2022)*

*Assessing Needs; Innovation Recipients (2022)*

**5) Was there anything that stopped you from attending groups?**

*Probes:*

- *Did you miss any CST sessions?*
  - *If so, what were the reasons for this?*
    - *What impact did missing these sessions have on your experience of CST?*
- *Costs*
  - *How much money was spent travelling to sessions?*
  - *How did you feel about the cost? Was it acceptable/bearable?*
  - *How might it cost less?*
- *Transport*
  - *Transport problems or carer unable to accompany to travel to CST sessions?*
  - *If you were to pay for transport, would you still attend CST sessions?*
  - *Where did you travel from? Alone or with a carer?*
- *Were there any events or religious gatherings (such as Easter) that affected your attendance for CST sessions?*
- *Do friends and family know that you have dementia?*
  - *If so, have you had any negative experiences because of this?*
- *Did people know you were attending CST?*
  - *Have friends and family reacted in a negative way to you attending CST?*
- *How did you feel about people knowing about your CST?*
  - *Did this stop you from attending?*
- *Did relationships with other participants in the group affect attendance?*

*CFIR Constructs:*

*Patient Needs and Resources (2009) Innovation Recipients; Capability/Opportunity/Motivation/Need (2022)*

*Cost (2009) / Innovation Cost (2022)*

*Available Resources (2009) / Available Resources; Funding (2022)*

*Local attitudes (2022)*

*Critical Incidents (2022)*

# Impact of COVID-19

1. **Do you think COVID-19 affected your experience of CST?**

*Probes:*

- *Any general worries about COVID-19? (mixing, hospital setting) How has COVID-19 affected your local community/ yourself?*
- *Whether people were wearing masks? Did this affect the delivery of CST?*

*CFIR Constructs:*

*Critical Incidents (2022)*

1. **What are your thoughts on conducting CST over the phone?**

*Probes:*

- *Do you have access to technology to do it virtually? (smartphone, computer/ tablet, data/ Wi-Fi)*
- *Would you need/ be able to borrow?*
- *Would you prefer to do in-person sessions or virtually from home? Why?*
- *What would be different about doing it virtually? Would anything be better/ worse?*

*CFIR Constructs:*

*Patient Needs and Resources (2009) Innovation Recipients; Capability/Opportunity/Motivation/Need (2022)*

*Relative Advantage (2009) / Innovation Relative Advantage (2009)*

*Adaptability (2009) / Innovation Adaptability (2022)*

# Overall Experience and Recommendations

1. **Generally speaking, what do you think of the CST programme?**

*Probes:*

- *What were the benefits for you?*
- *What did you not enjoy?*

1. **How helpful do you think CST has been for you? Why?**

*Probes:*

- *How are your symptoms now compared to before doing CST?*
- *Have you noticed any changes in yourself? From before CST to after?*
- *Do you feel any different since the sessions?*
- *Are you doing anything differently in your day-to-day life since the sessions?*
- *What? Why? How?*

1. **If you had the option, would you recommend continuing CST? (longer course) Why/ why not?**

*Probes:*

- *How do you feel about finishing the CST programme?*
- *Do you think CST was suitable for your needs? Was CST beneficial for you?*
  - *If not, why?*

1. **Would you recommend CST to other people in this region/ country? Why/ why not?**
2. **Do you have any suggestions for other people with dementia who have not yet started using CST?**
3. **How would you improve the CST programme? Why?**

*Probes:*

- *Any suggestions to make it more accessible to people?*

# Final Question

1. **Is there anything else you would like to tell us about your experience of CST?**

# End of Interview

Thank you very much for answering these questions. I will now turn the recorder off.

**END**

References

Damschroder, L. J., Aron, D. C., Keith, R. E., Kirsh, S. R., Alexander, J. A., & Lowery, J. C. (2009). Fostering implementation of health services research findings into practice: A consolidated framework for advancing implementation science. Implementation Science, 4(1). https://doi.org/10.1186/1748-5908-4-50

Damschroder, L. J., Reardon, C. M., Widerquist, M. A. O., & Lowery, J. (2022). The updated Consolidated Framework for Implementation Research based on user feedback. Implementation Science 2022 17:1, 17(1), 1–16. https://doi.org/10.1186/S13012-022-01245-0
